# Salivary lactate dehydrogenase and aminotransferases in diabetic patients

**DOI:** 10.1097/MD.0000000000005211

**Published:** 2016-11-28

**Authors:** Barbara Malicka, Katarzyna Skoskiewicz-Malinowska, Urszula Kaczmarek

**Affiliations:** Department of Conservative Dentistry and Paediatric Dentistry, Wroclaw Medical University, Poland.

**Keywords:** alanine aminotransferases, aspartate aminotransferases, diabetes mellitus, lactate dehydrogenase, saliva

## Abstract

Diabetes mellitus (DM) is a group of metabolic diseases resulting from impaired insulin secretion and/or action. DM is characterized by hyperglycemia that can lead to the dysfunction or damage of organs, including the salivary glands.

The aim of this study was to compare the levels of salivary lactate dehydrogenase (LDH), aspartate aminotransferase (AST), and alanine aminotransferase (ALT) in diabetic patients.

The study was approved by the Bioethics Committee of Wroclaw Medical University (Poland). The study comprised 90 adults of both sexes, aged 21 to 57 years. The patients were divided into 3 groups: type 1 diabetics (D1), type 2 diabetics (D2), and a healthy control group (C). Each group consisted of 30 age- and sex-matched subjects. Total protein (P, by Lowry method), LDH, AST, ALT (with Alpha Diagnostics kits), and salivary flow rate were measured in unstimulated mixed saliva. The level of glycosylated hemoglobin (HbA1c) was measured with DCA 2000 Reagent Kit. The obtained data were analyzed using the Mann–Whitney *U* test and the Spearman rank at a significance level of *P* < 0.05 with the use of STATISTICA 9.0 software.

In comparison with C, D1 presented a significantly higher activity of LDH (*P* < 0.001), AST (*P* < 0.001), and ALT (*P* < 0.01), whereas D2 indicated higher levels of LDH (*P* < 0.001) and ALT (*P* < 0.05) compared with C. Comparing D1 to D2, approximately 3-fold higher activity of AST (*P* < 0.01) and approximately 4.5-fold higher activity of ALT (*P* < 0.01) was observed.

Higher levels of salivary LDH, AST, and ALT in D1 compared with D2 and C confirm that salivary glands of D1 might be attributed to autoimmunological damage associated with the pathomechanism of DM.

## 1 Introduction

Diabetes mellitus (DM) is a group of metabolic disorders resulting from defects in insulin secretion and/or action that manifests itself with hyperglycemia leading to the dysfunction and damage of various organs, including the salivary glands. DM is classified into 4 categories: type 1 diabetes (destruction of pancreatic β-cells leading to insulin deficiency), type 2 diabetes (progressive insulin secretory defect), gestational diabetes (during pregnancy), and specific types of diabetes (due to other causes).^[1,2]^ It has been estimated that, on a global scale, diabetes may affect approximately 8.8% of the adult population for the year 2015.^[3]^

The long-lasting hyperglycemia that occurs in the course of DM leads to the formation of advanced glycation end products (AGEs) whose accumulation in plasma and tissues is associated with diabetic complications. As poorly controlled DM increases the risk of complications and blood glycated hemoglobin (HbA1c) is a valuable indicator used to determine average glucose plasma concentration during the past 2 to 3 months.^[4–6]^ The manifestations and complications of DM in the oral cavity include an increased risk of periodontal diseases (gingivitis and periodontitis), reduced salivary flow (xerostomia), changes in saliva composition, taste dysfunction, poor oral wound healing, increased risk of opportunistic *Candida albicans* infections, susceptibility to oral bacterial infections, increased occurrence of mucosal diseases, neurosensory oral disorder (burning mouth syndrome), recurrent dental caries, and tooth loss.^[7,8]^

It has been found that salivary glands in diabetic patients can be damaged leading to qualitative and quantitative changes in the saliva. However, the results of studies on the salivary flow rate are not always consistent. A lower flow rate of unstimulated saliva was observed in patients with type 1^[9–15]^ and type 2 diabetes.^[16]^ Other studies, however, did not reveal any differences in unstimulated and stimulated salivary flow rate in patients with type 1 diabetes^[17,18]^ and unstimulated salivary flow rate in patients with type 2 diabetes.^[15,19,20]^ Numerous salivary components have been studied in relation to DM, to its duration, and metabolic control. The following findings have been reported when diabetic patients were compared with the control group: lower pH,^[16,21]^ higher^[11–14,16]^ or lower total protein concentration,^[20,22]^ higher glucose level,^[12,13,16,20,22]^ higher^[21,23]^ or lower calcium level,^[13,16]^ higher sodium and potassium level,^[16]^ higher phosphate and magnesium levels,^[23]^ the same concentration of creatinine,^[23]^ higher urea,^[13]^ lower α-amylase,^[16,22]^ higher^[23–25]^ or the same^[26]^ levels of aspartate aminotransferase (AST) and alanine aminotransferase (ALT), higher^[14,23]^ or the same level of lactate dehydrogenase (LDH),^[26]^ higher level of salivary antioxidants,^[9]^ higher salivary IgA,^[12]^ the same level of salivary antimicrobial factors (except for salivary peroxidase),^[18]^ and increased myeloperoxidase activity and IgA content.^[15]^

Insulin-dependent diabetes (type 1) is a chronic specific disease caused by metabolic disorders of autoimmune origin.^[27,28]^ Type 1 diabetes is associated with other autoimmune diseases including the most frequent organ-specific autoimmune diseases such as autoimmune thyroid disease, celiac disease, autoimmune gastric disease.^[27,28]^ Markopoulos and Belazi^[29]^ studied the labial salivary glands taken from children at the onset of DM and observed glandular destruction (lymphocytic infiltration consisting mainly of T cells). The authors concluded that the similarity of glandular destruction to insulitis could suggest that the pancreas and salivary glands may share a common antigen that was the target of the autoimmune process. Markopoulos et al^[30]^ determined islet cell autoantibodies (ICA), endogenous insulin autoantibodies (IAA), glutamic acid decarboxylase autoantibodies (GADA), and tyrosine phosphatase autoantibodies (IA2A) as cell damage markers. The occurrence of GADA autoantibodies in the saliva of patients with type 1 diabetes can explain an autoimmune attack on the salivary glands.^[30]^ Moreover, a scintigraphic examination of the major salivary glands in patients with DM revealed impaired salivary secretion.^[31,32]^ Postmortem examination of submandibular glands from subjects with late onset of diabetes showed their enlargement due to fibrous tissue, fat, and vessels.^[33]^ Asymptomatic enlargement of parotid glands was observed in 24% of patients with DM.^[34]^ Secretion of saliva and its content can be altered by metabolic, nutritional, and neurological disturbances, hydration status, anticholinergics, antihistaminics, and diuretics.^[35]^ Salivary secretion can be affected by diabetes-related microvascular abnormalities and autonomic neuropathy. Poor glycemic control of DM leads to increased diuresis and fluid loss.^[35]^

Salivary activity of cytological enzymes such as AST and ALT, and LDH is a possible indicator of salivary gland involvement in the pathomechanism of DM.^[23]^ LDH is involved in anaerobic glycolysis that catalyzes pyruvate to lactic acid with NADH2 as an electron donor. LDH in mixed saliva comes from major and minor salivary glands (8.2% from parotid gland, 14.7% from submandibular and sublingual glands, 75% from gingival crevicular fluid, buccal epithelial cells, gastrointestinal reflux, and cell precipitate).^[36]^ The activity of isoenzymes profile of LDH is different in saliva and in the plasma. LDH4 and LDH5 enzymes dominate in saliva, whereas LDH1 and LDH2 dominate in the blood.^[36,37]^ LDH activity and the isoenzymes profile help monitor the course of different diseases (leukemia, myocardial infraction). Therefore, salivary LDH activity level has been used as a diagnostic tool for oral cavity cancer and as a biochemical marker of periodontal disease.^[37]^ ALT and AST are involved in protein metabolism by transferring amine groups from amino acids to alpha-keto acids. For diagnostic purposes, ALT and AST levels as well as De Ritis ratio (AST/ALT ratio) are checked in blood.

The aim of the study was to compare the levels of salivary LDH, AST, and ALT in diabetic patients.

## 2 Material and methods

The study was approved by the Bioethics Committee of Wroclaw Medical University (Poland).

### 2.1 Study participants

The study comprised 90 adults of both sexes, aged 21 to 57 years (Table 1). The patients were divided into 3 groups: type 1 diabetics (D1), type 2 diabetics (D2), and healthy control subjects (C). Each group consisted of 30 age- and sex-matched subjects. DM was diagnosed based on American Diabetes Association (ADA) criteria.^[1,2]^ Diabetic patients were treated at Wroclaw Medical University outpatient Diabetic Clinics, Poland. Patients fulfilled the following inclusion criteria: type 1 or 2 diabetes diagnosed at least one year before the study entry, no evidence of other systemic diseases, taking no medications that interfere with salivary secretion, and non-smoking.

**Table 1:**
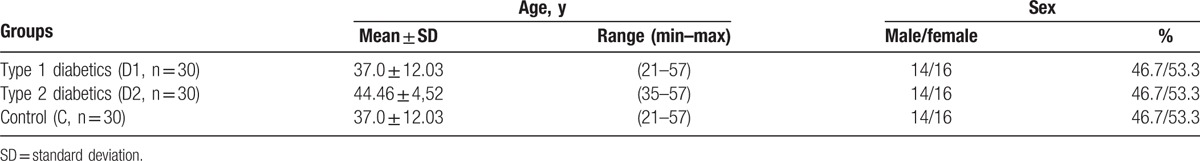
Characteristics of the study population.

The control subjects comprised generally healthy outpatients (Table 1) from the Department of Conservative and Paediatric Dentistry at Medical University, who had no evidence of systemic diseases, did not take any medications, and were non-smokers.

Two trained and calibrated dentists performed the examination. All patients signed informed consents. A prospective study was carried out for the period of 6 months. Diabetic patients were additionally divided into subgroups according to level of metabolic control. HbA1c = 8.5%, similar to the studies by Onishi et al,^[38]^ Yoon et al,^[39]^ Christie et al,^[40]^ and Cakmak et al,^[41]^ was regarded as a reference value. In this way 4 subgroups were created: with good metabolic control HbA1c ≤8.5%, subgroup D1-a, n = 14, mean HbA1c 7.45 ± 1.01%; subgroup D2-a, n = 14, mean HbA1c 7.37 ± 0.78% and poor metabolic control HbA1c >8.5%, subgroup D1-b, n = 16, mean HbA1c 9.75 ± 0.95%; subgroup D2-b, n = 15 mean HbA1c 9.45 ± 0.72%. Additionally, the diabetic patients were subdivided with regard to the duration of DM ≤10 years (subgroup D1-c, n = 14, mean 6.13 ± 3.26 years; subgroup D2-c, n = 14, mean 5.51 ± 2.94 years), and >10 years (subgroup D1-d, n = 16, mean 19.63 ± 7.35 years; subgroup D2-d, n = 16, mean 17.66 ± 4.79 years).

The saliva was collected after thoroughly rinsing the mouth with distilled water. Saliva is a complex biological fluid, a mixture of secretions from the major and minor salivary glands, constituents of non-salivary origin (i.e., gingival crevicular fluid, desquamated epithelial cells, microorganisms and their products, leukocytes), and extrinsic substances (e.g., food debris). The aim of collecting salivary samples after rinsing the oral cavity and using clear supernatants for the assays was to minimize the contribution of other sources of the tested enzymes such as oral bacteria, epithelial cells, leukocytes, and dietary residues. Therefore, we assume that a major part of the examined salivary constituents comes from salivary glands.^[26,29]^

Unstimulated mixed saliva was collected at least 1 hour after the patients’ usual breakfast, and after thoroughly rinsing the mouth with distilled water. The patients were sitting with their head bent down and their mouth open. Approximately 5 mL of saliva was collected with a plastic pipette, and the sample was put into a graded test tube stored on crushed ice. Based on the volume of the collected salivary sample and the time of its collection, salivary flow rate was calculated as mL/min (V). Salivary samples were centrifuged for 10 minutes at a speed of 3400 rpm before biochemical assays.

Total protein (P, by Lowry method), lactate dehydrogenase (LDH, EC 1.1.1.27), aspartate aminotransferase (AST, GOT, EC 2.6.1.1), and alanine aminotransferase (ALT, GPT, EC 2.6.1.2) were determined in the samples. Enzymes were assessed using Alpha Diagnostics kits (Warsaw); LDH by the method in which the formation rate of NADH due to the LDH oxidation reaction of l-lactate to pyruvate was measured; AST by the method based on transfer of an amino group from l-aspartate to 2-α-ketoglutarate with formation of oxaloacetate and l-glutamate and next oxaloacetate was reduced to malate by malate dehydrogenase with simultaneous oxidation of NADH to NAD+; ALT, similar to AST, by the method based on transfer of an amino group from l-alanine to 2-α-ketoglutarate with formation of pyruvate which was reduced next to l-lactate by LDH with simultaneous oxidation of NADH to NAD+. The total protein concentration was expressed as mg/mL and output (mg/min), the activity of the enzymes as mIU/mL, specific activity (mIU/1 mg protein) and output (mIU/min). Moreover, in diabetic patients, simultaneous to the salivary samples collection, the level of HbA1c was measured with the use of the standard method (DCA 2000 HbA1c Reagent Kit, Siemens, Simens/Bayer).

### 2.2 Clinical examination

The periodontal condition was assessed with the use of Periodontal Screening and Recording Index—PSR index.^[42]^

### 2.3 The statistical analysis

The obtained data were analyzed with the use of Mann–Whitney *U* test, χ^2^ test, and Spearman rank correlation at a significance level of *P* < 0.05 with the help of STATISTICA 9.0 software (StatSoft Polska). To describe the data, mean ± SD was used. The differences between the mean values of the salivary parameters in patients with diabetes type 1, 2 and in the control group, as well as in subgroups of patients with different level of metabolic control and duration of disease were compared using the Mann–Whitney *U* test. The periodontal parameters were assessed with the use of χ^2^ test. In addition, Spearman rank correlation was used to determine the relationship among the enzyme levels, salivary flow rate, and level of HbA1c.

## 3 Results

Significantly lower salivary flow rate (approximately 34%, *P* < 0.001), higher levels of LDH (*P* < 0.001), AST (*P* < 0.001), and ALT (*P* < 0.01), higher levels of specific activities of LDH (*P* < 0.05) and AST (*P* < 0.05) in D1 compared with C were observed. Lower salivary flow rate (approximately 23%, *P* < 0.05), higher levels of LDH (*P* < 0.00001) and ALT (*P* < 0.05), higher levels of specific activities of LDH (*P* < 0.01) and ALT (*P* < 0.05) in D2 compared with C were noticed. Comparing D1 with D2, approximately 3-fold higher activity of AST (*P* < 0.01) and approximately 4.5-fold higher activity of ALT (*P* < 0.01) was found (Table 2).

**Table 2:**
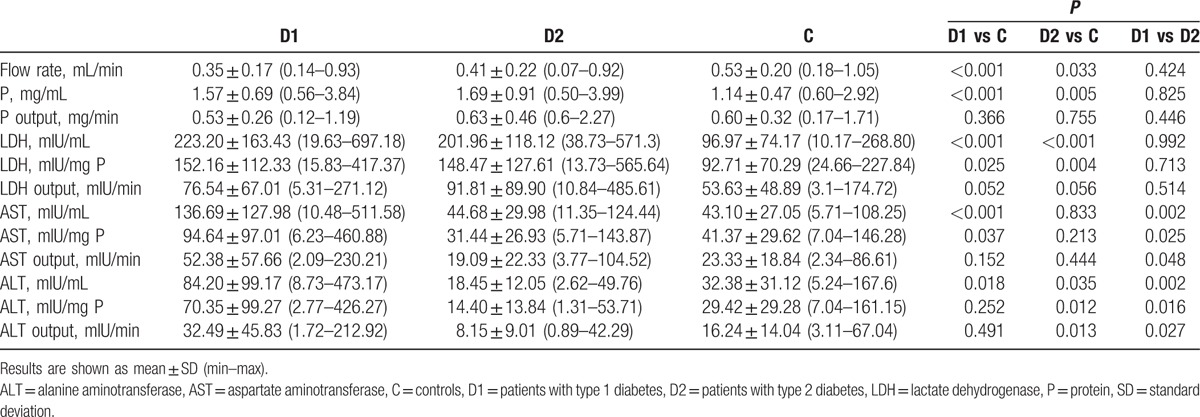
Salivary parameters in diabetics and healthy subjects.

When comparing subgroups of diabetics which were formed with regards to the control of the disease, no significant difference between D1-a and D1-b, and D2-a and D2-b was found (Table 3). Having compared D1 or D2 with regard to disease duration, we observed no significant differences, except for an approximately 2-fold higher level of salivary AST in D2-d (*P* < 0.05) (Table 4).

**Table 3:**
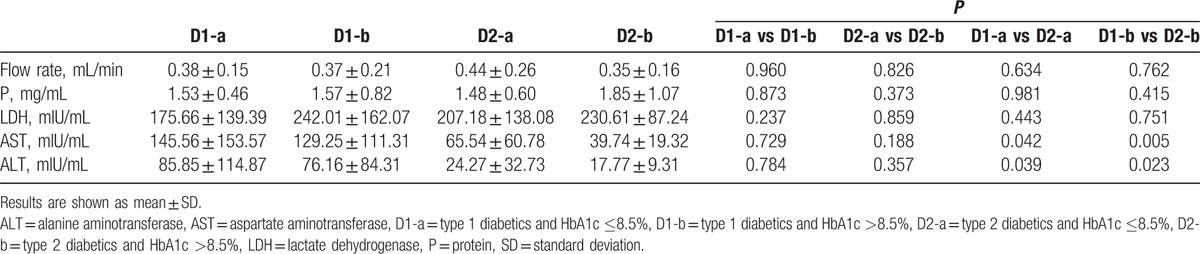
Salivary parameters in subgroups of diabetics with well and poor metabolic control of the disease.

**Table 4:**
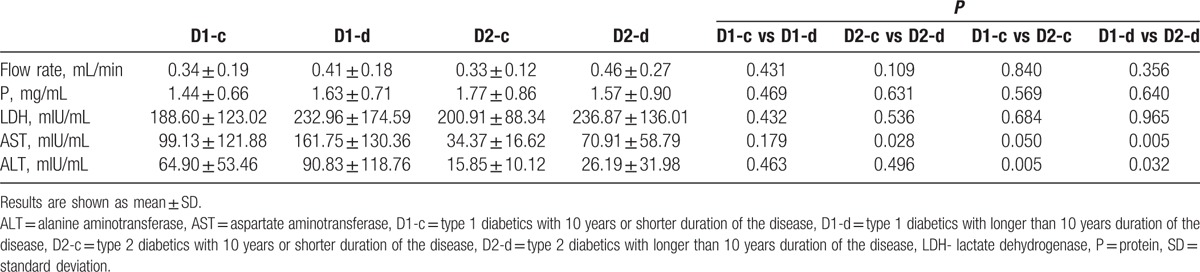
Salivary parameters in subgroups of diabetics with shorter or longer duration of the disease.

A positive correlation between ALT and HbA1c (*r* = 0.49, *P* = 0.04) in D1-b was found. No correlation between salivary enzymes and salivary flow rate was noticed. A negative correlation between ALT and the total protein concentration in D1 (r = −0.38, *P* = 0.03) was observed. Moreover, a positive correlation between salivary ALT and AST in diabetic patients and in healthy control (D1 group *r* = 0.59, *P* = 0.001; D2 group *r* = 0.37, *P* = 0.05; control group *r* = 0.51, *P* = 0.003) were registered.

Periodontal condition evaluated with the use of PSR in both diabetic groups was worse compared with healthy control, which was manifested by the higher percentage of shallow and deep periodontal pockets (Table 5).

**Table 5:**
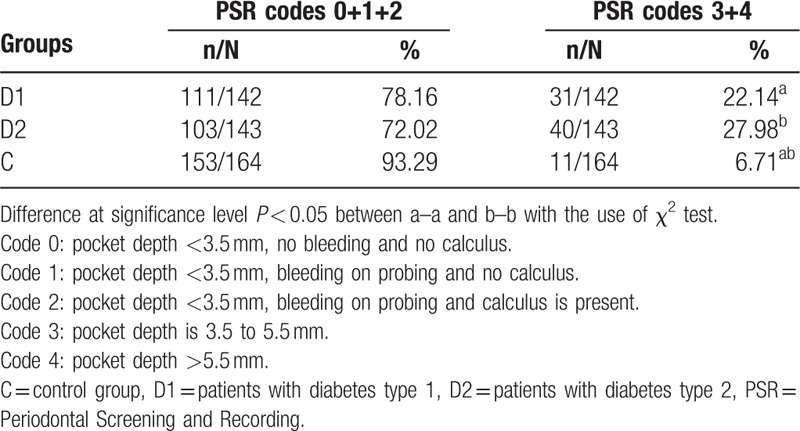
Comparison of the distribution of PSR codes in patients with diabetes and healthy subjects.

## 4 Discussion

Normal unstimulated salivary flow rate ranges from 0.3 to 0.5 mL/min, and flow rates between 0.10 and 0.01 mL/min are considered to be hyposalivation. Our data present the mean values of unstimulated mixed saliva flow rate in diabetic patients as within the range of normal secretion. The salivary flow rate in D1 and D2 was significantly diminished when compared with the control group (approximately 34% and 23%, respectively). The obtained data are consistent with the results obtained in children with type 1 diabetes^[9,11–14,21]^ and adults with type 2 diabetes.^[16,22]^ We found no significant differences in the salivary flow rate between D1 and D2 with regard to the level of metabolic control, similar to the data obtained in diabetic children.^[11,14]^ Comparing D1 and D2 with regard to disease duration, no significant differences in salivary secretion were noticed, similar to children with type 1 diabetes.^[14]^ Glycosuria, triggered by even small hyperglycemia, can reduce salivary flow in diabetic patients and lead to liquid loss and dehydration, and, consequently, to the salivary flow reduction.^[43]^ Moreover, it cannot be ignored that DM has a negative effect on the nervous system and that it develops microangiopathy and hormone imbalances leading to xerostomia.^[44]^

Total protein concentration was higher in D1 and D2 when compared with C. These data are consistent with the data obtained in children with type 1 diabetes^[13,14]^ and adults with type 2 diabetes.^[16]^ Our data showed significantly higher activities of salivary AST and ALT in D1 compared with C. Similar data concerning adult subjects were presented by Musumeci et al,^[23]^ Al-Rubee et al,^[24]^ and Verma et al.^[25]^ However, Cinquini et al^[26]^ did not notice any significant differences in the level of salivary AST and ALT in children with type 1 diabetes compared with the control group. Verma et al,^[25]^ who reported increased activity levels of salivary AST and ALT in patients with type 1 diabetes compared with patients with type 2 diabetes and to the healthy control, obtained similar results. In patients with type 1 diabetes, significantly higher activities of LDH, AST, and ALT compared with the healthy control and higher activity of AST and ALT compared with patients with type 2 diabetes were found.^[25]^ D1 demonstrated almost 3-fold higher activity of AST and ALT than in the healthy group. A comparison of D1 with D2 revealed significant higher activity of AST and ALT in all subgroups regardless of the disease control and its duration. A significantly higher LDH value in the subgroup with a shorter duration of the disease by Cinquini et al^[26]^ and Kaczmarek and Mysiak-Dębska^[14]^ was noticed. The level of metabolic control was not reflected in those enzyme values in type 1 and type 2 diabetic patients, or in children suffering from diabetes.^[14]^

LDH, ALT, and AST are located in the cytoplasm of cells (AST is also present in mitochondria), and are widely distributed in tissues. The increased activity level in serum is caused by their leakage from damaged tissues. Their increase in saliva may, in turn, result from damage to salivary glands cells caused by numerous mechanisms.^[23,26,36]^ Musumeci et al^[23]^ observed that increased activity of AST, ALT, and LDH in saliva of adults with type 1 and 2 diabetes is associated with salivary gland cells damage. Cinquini et al,^[26]^ however, on the basis of a histopathological examination of salivary glands in children with type 1 diabetes, registered that lymphocytic infiltration causes cell damage. Therefore, it was postulated that salivary glands are subjected to the activity of autoimmune cells by direct action of antibodies against beta cells of pancreatic islets or specific antibodies against salivary gland antigens.^[26]^ This hypothesis was supported by Markopoulos et al^[30]^ noticed high levels of glutamic acid decarboxylase (GAD) antibodies in blood and saliva of children with type 1 diabetes against beta cell antigens, which indicates that submandibular duct cells may be the target of autoimmune attack against submandibular gland. We found that in all studied groups the salivary AST activity was positively correlated with ALT, similar to what was observed by Musumeci et al.^[23]^ Furthermore, it was observed that a positive correlation exists between salivary ALT level and HbA1c in DM1 patients with poor glycemic control. These findings would indirectly confirm the hypothesis that inappropriate disease control promotes the formation of diabetic complications, including complications in the oral cavity. Some authors observed the linear relationship between HbA1c levels and the risk of complications.^[41,45]^ Cakmak et al^[41]^ suggested that HbA1c level is prognostic factor associated with mortality after acute myocardial infarction. It should be emphasized that lower HbA1c value obtained by intensive diabetes treatment reduces the risk of diabetes complications such as stroke, myocardial infarction, neuropathy, and others.^[46]^ The salivary levels of AST and ALT seemed to be unrelated to the duration of the disease, since the data obtained in this study and by Musumeci et al^[23]^ did not reveal such a correlation. However, after examining type 1 diabetes in children, Cinquini et al^[26]^ reported a negative correlation between levels of salivary AST and ALT and the duration of the disease.

Taking into consideration no lack of relation between enzyme activity and metabolic control of the disease, Verma et al^[25]^ suggested that damage to the salivary glands in patients with type 1 diabetes can be caused by mechanisms other than autoimmunity. Therefore, we cannot ignore the fact that the inflammatory process in periodontal tissues can increase the activity of cytoplasmic enzymes in saliva.^[47,48,49]^ Ikekpeazu et al^[49]^ reported that periodontitis and co-occurrence of diabetes lead to increases in salivary LDH, AST, and ALT activity. In our study, we found worse periodontal condition expressed by PSR index in D1 and D2 with respect to C. More advanced periodontal lesions in D2 compared with D1 (*P* > 0.05) were observed. By comparing these data to the studied salivary enzyme levels, we can hypothesize that periodontal inflammation and probable autoimmune processes significantly increase the levels of salivary enzymes in D1 compared with D2 and C.

## 5 Conclusion

Within the limitation of the study, it can be suggested that higher levels of salivary LDH, AST, and ALT in diabetics are related to the salivary gland involvement in the course of DM. The higher activity of salivary LDH, AST, and ALT in D1 compared with D2 and C can confirm the hypothesis that salivary glands in D1 might be attributed to autoimmune damage associated with the pathomechanism of DM.
